# Assessing and optimizing Team-Based Learning in undergraduate cosmetic dermatology education: an empirical study using interpretable machine learning

**DOI:** 10.1186/s12909-025-08325-x

**Published:** 2025-12-23

**Authors:** Pingxiang Ouyang, Lu Zhou, Lihua Gao, Jianyun Lu, Jinrong Zeng

**Affiliations:** Department of Dermatology, The Third Xiangya Hospital, Central South University, 138 Tongzipo Road, Changsha, China

**Keywords:** Cosmetic dermatology, Team-Based learning, Artificial intelligence, Machine learning, SHapley additive explanations, Teaching reform

## Abstract

**Background:**

The research aimed to explore the application effectiveness, advantages, and disadvantages of Team-Based Learning (TBL) in undergraduate cosmetic dermatology education and to analyze directions for pedagogical improvement using interpretable machine learning (IML).

**Methods:**

A total of 154 undergraduate clinical medicine students from Xiangya School of Medicine, Central South University were included as study subjects. Using the cosmetic dermatology chapter (two teaching units) from the Dermatology and Venereology course, a comparative design was implemented between traditional Lecture-Based Learning (LBL) and interest-oriented TBL. Teaching feedback was collected through questionnaires, and IML and correlation analysis were applied to identify key nodes for pedagogical optimization.

**Results:**

Surveys showed that 83.8% of students reported increased learning interest, 84.4% were willing to engage in post-class self-directed learning, 81.2% were satisfied with the course, and 86.4% desired more TBL courses. Recognition of teaching components revealed that 87.0% preferred the presentation part, 89.0% endorsed the teamwork part, 80.5% agreed with the questioning part, and 95.5% valued the discussion part. Compared to LBL, TBL significantly improved self-directed learning capability (*P* < 0.001) and knowledge mastery capability (*P* = 0.004), but showed weaker improvement in independent thinking capability (*P* = 0.028). IML revealed that the most important feature determining the enthusiasm for cosmetic dermatology and TBL was the enhancement of independent thinking capability. Course satisfaction was primarily influenced by improved self-directed learning capability. Correlation analysis indicated that the recognition of most teaching components positively correlated with learning interest in cosmetic dermatology and satisfaction with TBL, except that students with higher recognition of the Teamwork Part (*r* = -0.432) and Questioning Part (*r* = -0.900) paradoxically exhibited resistance to the implementation of additional TBL courses.

**Conclusion:**

TBL effectively enhances students’ learning interest and comprehensive competencies in cosmetic dermatology education, demonstrating strong potential for broader application. However, significant opportunities for refinement persist in instructional design.

## Introduction

The skin, as the largest organ of the human body [1, 2], directly reflects an individual’s physiological health and psychological state through its appearance. With continuous societal development, the demand for cosmetic dermatology has grown exponentially [3–5], propelling cosmetic dermatology to become a critical subspecialty within modern dermatology and venereology, now integrated into undergraduate medical curricula at many institutions. However, the current undergraduate medical education system faces a structural contradiction: compared to other clinical disciplines, dermatology and venereology possess a broad disease spectrum and require highly specialized expertise, yet they have long been allocated significantly fewer teaching hours and credits than core disciplines such as internal medicine and surgery in medical curricula [6, 7]. While this allocation might have been justifiable in the past when dermatologic health demands were relatively limited, it is no longer adequate to address the growing demands for skin disease prevention, treatment, and health management today. This contradiction compels dermatology and venereology education to prioritize skin diseases with significant health impacts, thereby marginalizing cosmetic dermatology. Consequently, developing innovative pedagogical models that transcend the constraints of limited teaching hours and effectively stimulate learning motivation is not only an urgent necessity for advancing cosmetic dermatology but also addresses the needs of other similarly marginalized disciplines.

Team-Based Learning (TBL), proposed by American educational theorist Professor Larry Michaelsen and colleagues, is a novel pedagogical model centered on structured team collaboration. Compared to traditional Lecture-Based Learning (LBL), TBL demonstrates significant advantages in enhancing learning motivation, strengthening team collaboration efficacy, and fostering self-directed learning capabilities [8, 9]. This model transforms the instructor’s role from a traditional knowledge disseminator to a facilitator and coordinator of the learning process, shifting pedagogy from unidirectional knowledge transfer to multidimensional interactive discussion. Despite its global adoption in education, TBL retains inherent limitations that hinder its ability to displace LBL’s dominance in core curricula. Thus, adaptive refinement of TBL through pedagogical innovation holds practical value for expanding its application across disciplines.

The application of Artificial Intelligence (AI) in dermatology continues to expand, progressing from initial AI-assisted dermatological image analysis to disease risk prediction, health monitoring and management, and further extending to critical areas such as medical education and clinical practice [10–12]. In the current educational landscape, most studies on AI applications remain limited to using generative AI for producing suggestions for various teaching activities [13, 14]. However, the lack of content authenticity verification mechanisms in generative AI introduces potential risks. This study innovatively employs supervised Interpretable Machine Learning (IML) to assess the teaching outcomes of TBL within the traditional LBL framework, aiming to systematically evaluate the feasibility of TBL in undergraduate cosmetic dermatology education. Through feature importance analysis, we identify core factors influencing TBL implementation and propose targeted improvement strategies, thereby providing evidence-based guidance for optimizing novel pedagogical models.

### Team-based learning

TBL, proposed by American educationalist Professor Larry Michaelsen and colleagues, is a novel pedagogical approach characterized by team collaboration, initially mainly used in business courses [15]. Its standardized implementation process comprises three phases: (1) Pre-class Preparation Phase: Learners complete knowledge acquisition through literature review and self-directed study; (2) Team Testing Phase: Teams collaboratively complete pre-class assessments to ensure capability for participation; (3) Team Application Phase: With scenario simulations and case discussions, teams generate solutions within time constraints and address cross-group inquiries [16]. This three-dimensional framework, “self-construction, collaborative validation, practical application”, ensures active participation of all students, enhancing engagement, initiative, and satisfaction [17–19]. Consequently, TBL has been increasingly trialed in medical education [20, 21], particularly in peripheral disciplines.

Compared to traditional LBL, TBL demonstrates superior efficacy in boosting student motivation. Active participation correlates with significant improvements in academic performance and course satisfaction [22, 23]. Relative to Problem-Based Learning (PBL) and Case-Based Learning (CBL), TBL exhibits greater inclusivity: on one hand, a single instructor can guide multiple groups simultaneously within one classroom [24]; on the other, problem discussions and case analyses can be flexibly integrated into team collaboration [25]. In medical education, TBL’s unique value lies in cultivating healthcare professionals’ ability to resolve clinical challenges through interdisciplinary teamwork [26]– [27].

Despite its advantages, TBL faces core challenges in implementation. First, faculty development is a critical constraint. Unlike LBL’s unidirectional knowledge delivery, TBL demands multifaceted instructor competencies, including pedagogical material design, progressive problem development, and dynamic feedback management [28]. Thus, comprehensive TBL training for educators is paramount. Second, procedural complexity impacts effectiveness. While TBL enhances comprehensive competencies, its multi-phase design increases study load (e.g., time invested in teamwork and case discussions), which may lead to adaptation difficulties, necessitating context-driven simplification [27]. To address these challenges, existing strategies advocate selective modification of TBL phases while retaining core elements [29]. However, such adjustments often rely on educators’ subjective judgment, risking decision bias and deviation from learner-centered principles. Therefore, establishing an objective evaluation system based on multidimensional learning analytics is urgently needed to scientifically guide adaptive TBL optimization.

### Machine learning and feature importance evaluation

With the rapid advancement of computer technology, AI has become a core domain of contemporary technological development. As a critical branch of AI [30], machine learning is essentially the autonomous learning of algorithmic systems from data features to identify complex patterns that are difficult for humans to directly analyze and to enable predictive functions [31, 32]. Its technical implementation comprises three key phases: first, iterative learning on training datasets using predefined algorithms to build predictive models; second, validating model performance and optimizing parameters via testing datasets; and finally, applying optimized models to new data for predictive decision-making [33]. This data-driven approach not only uncovers hidden data laws but also enables precise outcome predictions for unknown samples [34], providing effective decision support for humans.

Current mainstream machine learning algorithms include decision trees, naive Bayes, support vector machines, neural networks, and ensemble methods derived from these base algorithms (e.g., random forests, XGBoost). According to the “no free lunch” (NFL) theorem, different algorithms exhibit distinct advantages in solving specific problem types [35], necessitating context-driven selection. For example, neural networks demonstrate powerful predictive capabilities by simulating neuronal signal transmission mechanisms [36] and excel in processing complex data such as images [37], but these advantages often come at the cost of model interpretability [32]. Ensemble algorithms generally achieve superior performance by integrating multiple models, though improper ensemble strategies may degrade performance, a phenomenon rarely systematically reported in existing studies.

While predictive performance remains a core evaluation metric for machine learning, interpreting the contribution of key features to predictions (feature importance ranking) has become a critical research focus [38–40]. However, most complex machine learning algorithms (e.g., deep neural networks) are inherently “black-box” models: users input data and receive predictions but cannot trace the internal logic of decision pathways. Notably, breakthroughs in IML have recently emerged, with novel explanation frameworks effectively demystifying model black boxes [38, 40, 41]. Existing IML methods can be categorized into three dimensions: (1) temporal interpretability: intrinsic or post-hoc explanations; (2) scope of explanation: global or local explanations; (3) model dependency: model-specific or model-agnostic explanations [42]. Among these, the SHapley Additive exPlanations (SHAP) method, based on Shapley values from game theory, quantifies the marginal contribution of each feature to predictions [43, 44], showing robust explanatory power.

In recent years, AI applications in education have expanded from basic pedagogical support (e.g., personalized learning plans, academic performance prediction, dynamic teaching strategy adjustment) to deeper theoretical constructs [45, 46]. Building on previous research, this study aims to elevate machine learning’s role in educational research by proposing a novel analytical framework: not only focusing on micro-level interventions in teacher-student behaviors but also leveraging machine learning to deconstruct core pedagogical elements and construct a quantifiable educational theory system. This “micro-macro” dual-layer research paradigm will drive the transformation of education from experience-driven practices to data-driven theoretical innovation, significantly expanding the disciplinary impact of educational research.

### Research objectives

Core objectives of this study include three dimensions:

- Empirically evaluating the feasibility of TBL in enhancing student engagement and pedagogical efficacy in peripheral disciplines such as cosmetic dermatology;
- Systematically analyzing the key impacts and mechanisms of TBL’s instructional components and outcomes on student acceptance using IML, with emphasis on identifying core teaching elements with the highest feature importance weights;
- Diagnosing existing core challenges in TBL implementation and proposing targeted improvement strategies based on SHAP attribution analysis, ultimately developing transferable optimization frameworks for teaching reform in marginalized disciplines.

## Methods

### Research design and participants

This study enrolled 154 undergraduate clinical medicine students from the teaching reform project of Xiangya School of Medicine, Central South University, all of whom were taught by the Department of Dermatology and Venereology at Third Xiangya Hospital. Two specialized lectures on cosmetic dermatology were conducted: one lecture for LBL, the other for TBL. These lectures cover fundamental therapeutic content, including pharmacotherapy, photoelectric therapy, injection therapy, and other modalities. This teaching activity deviated from conventional formats by implementing innovative structural design. As the activity involved human participants, the study protocol obtained prior approval from the Institutional Review Board of the Third Xiangya Hospital, Central South University (approval #: 24177), with written informed consent secured from all participants. All participants were aged 18 or above, voluntarily joined the research project, and experienced no physical or psychological harm throughout the activity. Participants engaged in an alternative approach to knowledge acquisition during this process.

### TBL teaching method

Building on prior TBL frameworks [47–49], we developed an interest-oriented TBL model with the following implementation steps: (1) Based on students’ self-preferences and cognitive heterogeneity analysis, each class was divided into 4–5 balanced teams of 4–5 members; (2) One week prior to class, theme-based pre-learning tasks covering core modules (e.g., laser technology, injectable cosmetics) were assigned. Teams selected research topics through consensus and completed evidence-based literature reviews and multimedia presentation materials preparation under instructor guidance; (3) During the in-class phase, a “student-centered, faculty-supervised” approach was adopted. Each team delivered a 30-minute thematic presentation (including cross-group Q&A sessions), with instructors documenting contentious issues in real-time and systematically addressing them during the summary session.

### Outcome measure

An online in-class test was conducted before each class to compare the differences in teaching effectiveness between TBL and LBL in enhancing self-directed learning capability. Another online in-class test was conducted at the end of the class to examine the differences in teaching effectiveness between TBL and LBL in improving three dimensions, independent thinking capability, clinical reasoning capability, and knowledge mastery capability. These test components cover multiple dimensions, including basic knowledge evaluation, clinical case analysis, and humanistic care competency assessment. All test results were independently scored by two professors, with the final score representing the average of their assessments. The results were standardized using a 10-point Likert scale (1 = minimal improvement, 10 = significant improvement). After the classes, an anonymous online survey system was used to collect students’ comprehensive evaluations of the TBL course: (1) General Teaching Process Evaluation: This included four indicators, improved interest in cosmetic dermatology, willingness for post-class extended learning, satisfaction with the teaching process, and demand for additional TBL courses, measured by binary response scales (yes/no); (2) Part-Specific Teaching Evaluation: This focused on assessing recognition of four core parts, presentation part, teamwork part, questioning part, and discussion part, also using binary scales.

### Statistical analysis

Continuous variables were described as median [Q1–Q3], and categorical variables as frequency [n (%)]. Since the in-class test scores under TBL and LBL modes demonstrated non-normal distribution (per Shapiro-Wilk test) and unequal variances (per Levene’s test), the Wilcoxon rank-sum test was employed in this study to compare the effectiveness of the two instructional approaches, with statistical significance defined as two-tailed *P* < 0.05.

To analyze the impact of teaching parts and capability improvements on evaluations, a machine learning framework was constructed: (1) This study partitioned the dataset randomly into training and testing sets at a 7:3 ratio, utilizing decision trees as base learners and constructing an ensemble model based on the Extreme Gradient Boosting (XGBoost) algorithm. XGBoost, an optimized distributed gradient boosting framework, builds upon traditional gradient boosting and tree model hyperparameters by incorporating additional hyperparameter configurations that help mitigate overfitting risks, thereby enhancing predictive accuracy. Furthermore, the algorithm supports early stopping and parallel processing, ensuring both reliable outcomes and efficient, flexible computation [50, 51]; (2) To distinguish IML from generative AI models with insufficient content authenticity verification, this study employed a 10-fold cross-validation approach to evaluate model performance, with the Area Under the Receiver Operating Characteristic (ROC) Curve (AUC) as the primary metric, preventing biased conclusions due to suboptimal model performance [52]; (3) The SHAP method was employed to interpret feature importance in the optimal XGBoost model. Based on game theory principles, SHAP decomposes model predictions into contributions from individual features, quantifying both the magnitude and direction of each feature’s influence on the model output. This approach not only explains the contribution of each feature in individual predictions but also identifies the most influential feature variables for overall model performance by aggregating SHAP values across multiple samples, thereby providing theoretically grounded interpretability for model predictions [43, 44]. Finally, Pearson correlation analysis was employed to validate the linear relationship between feature values and SHAP values, thus providing objective statistical evidence from the machine learning model to explain how various features influence TBL outcomes [53].

All analyses were conducted using SPSS (version 27.0.1) and R (version 4.4.2), with key packages including “lawstat” (version 3.6), “xgboost” (version 1.7.8.1), “caret” (version 7.0–1), “shapviz” (version 9.7).

## Results

### Overall recognition of TBL and recognition of each teaching part

**Fig. 1:**
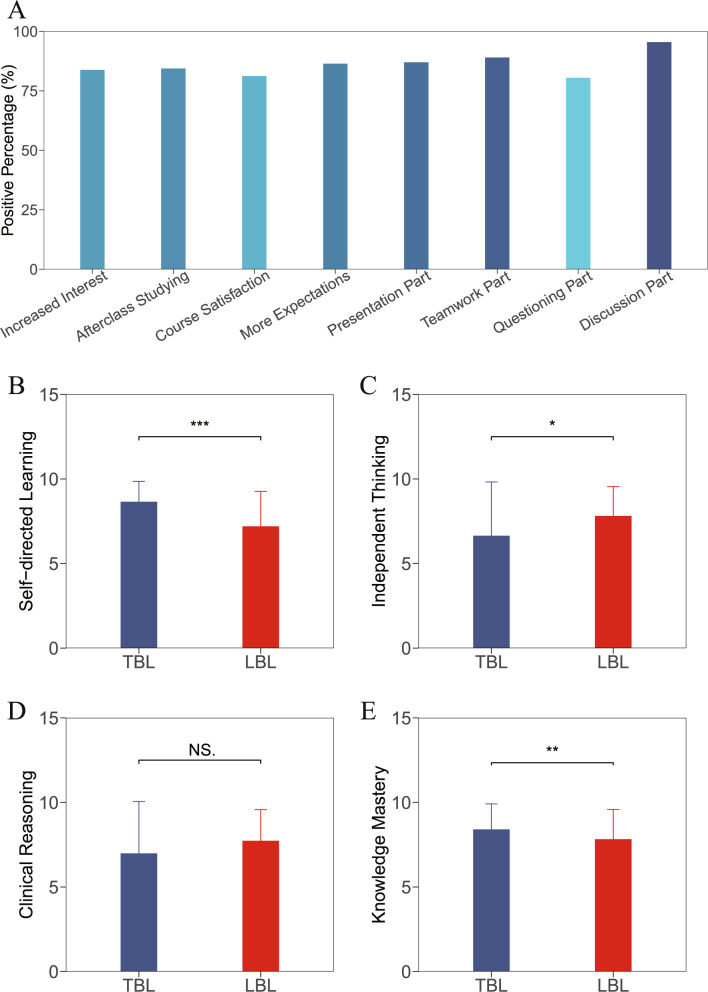
Educational feedback outcomes (**A**) Students’ overall attitude towards the TBL course and whether they enjoy each teaching part. **B**-**E** Students’ ratings of the degree to how TBL and LBL courses have helped improve their capabilities. NS.: *P* ≥ 0.05, *: *P* < 0.05, **: *P* < 0.01, ***: *P* < 0.001

This study achieved a 100% questionnaire response rate (*n* = 154), with aggregated results presented in Table 1. As shown in Fig. 1A, under the general teaching process evaluation dimension, 83.8% of students reported that TBL significantly enhanced their interest in cosmetic dermatology, 84.4% expressed willingness for post-class deep learning, 81.2% were satisfied with the teaching process, and 86.4% desired additional TBL courses. In part-specific evaluations, presentation part (87.0%), teamwork part (89.0%), questioning part (80.5%), and discussion part (95.5%) all received high recognition. Notably, approval for the discussion component exceeded 95%, highlighting the central value of interactive learning models. Students’ strong endorsement of teamwork part and discussion part indicates that, compared to independent learning, most learners prefer interactive and collaborative approaches for knowledge construction and problem-solving.

**Table 1:** Summary of all results

Questionnaire Questions	Results	*P*-value
Positive Increased Interest, n (%)	129 (83.8)	**-**
Positive Afterclass Studying, n (%)	130 (84.4)	**-**
Positive Course Satisfaction, n (%)	125 (81.2)	**-**
Positive More Expectations, n (%)	133 (86.4)	**-**
Enjoying Presentation Part, n (%)	134 (87.0)	**-**
Enjoying Teamwork Part, n (%)	137 (89.0)	**-**
Enjoying Questioning Part, n (%)	124 (80.5)	**-**
Enjoying Discussion Part, n (%)	147 (95.5)	**-**
Improving Self-directed Learning Capability (TBL), Median [Q1-Q3]	9 [8–10]	0.000
Improving Self-directed Learning Capability (LBL), Median [Q1-Q3]	7 [6–9]	
Improving Independent Thinking Capability (TBL), Median [Q1-Q3]	8 [6–9]	0.028
Improving Independent Thinking Capability (LBL), Median [Q1-Q3]	8 [7–9]	
Improving Clinical Reasoning Capability (TBL), Median [Q1-Q3]	8 [6–9]	0.528
Improving Clinical Reasoning Capability (LBL), Median [Q1-Q3]	8 [7–9]	
Improving Knowledge Mastery Capability (TBL), Median [Q1-Q3]	9 [7–10]	0.004
Improving Knowledge Mastery Capability (LBL), Median [Q1-Q3]	8 [7–9]	

Between-group comparisons are performed using the Wilcoxon rank-sum test, with *P* < 0.05 considered statistically significant

### Comparison of teaching efficacy between TBL and LBL

Since the capability improvement scores derived from test results exhibited non-normal distribution and heteroscedasticity; thus, the Wilcoxon rank-sum test was applied for group comparisons. As shown in Fig. 1B-E, Results indicated that TBL significantly outperformed LBL in enhancing self-directed learning capability (*P* < 0.001) and knowledge mastery capability (*P* = 0.004), while LBL showed greater efficacy in fostering independent thinking capability (*P* = 0.028). No statistically significant difference was observed between the two methods in improving clinical reasoning capability (*P* = 0.528). These results suggest that while TBL positively stimulates learning engagement and enhances knowledge comprehension, it may have limitations in fostering independent thinking skills, which requires attention and refinement.

### Influence of teaching part and capability improvement on students’ evaluation of TBL

**Fig. 2:**
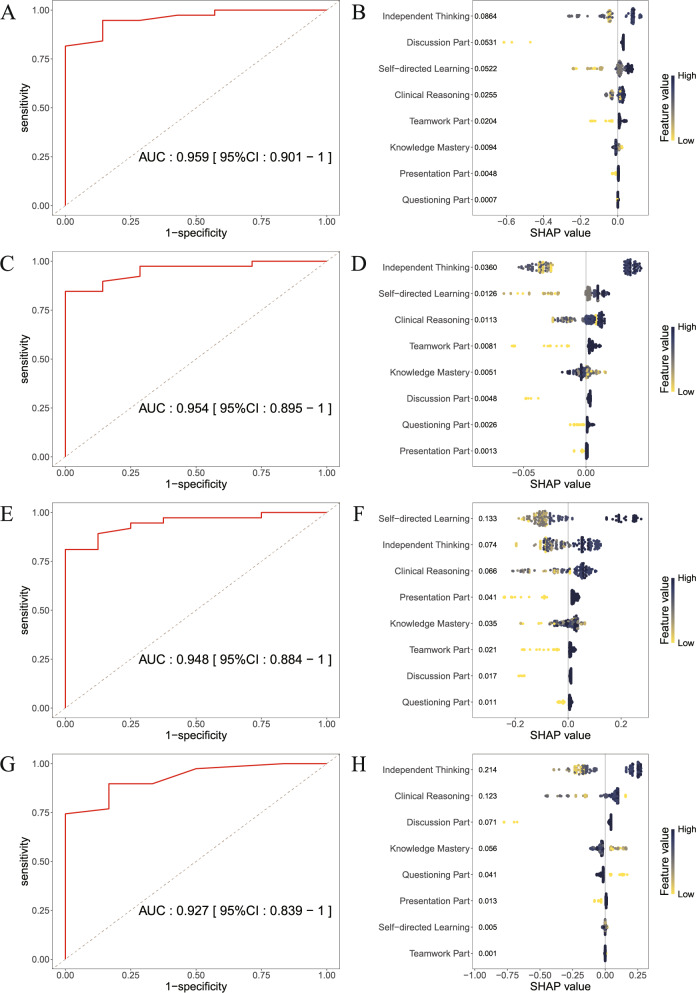
ROC curves and SHAP plots of XGBoost for predicting of students’ overall attitude towards the TBL course (**A**-**B**) Accuracy and feature importance ranking of the model predicting whether to enhance student interest; (**C**-**D**) Accuracy and feature importance ranking of the model predicting whether students will further learn after class; (**E**-**F**) Accuracy and feature importance ranking of the model predicting students’ satisfaction with this course; (**G**-**H**) Accuracy and feature importance ranking of the model predicting whether students expect more TBL courses

Based on a large subset of randomly selected training data, XGBoost ensemble models were constructed to predict core teaching metrics using the students’ recognition of TBL parts (presentation part, teamwork part, questioning part, discussion part) and the capability improvement scores (self-directed learning, knowledge mastery, independent thinking, clinical reasoning) as feature variables. Through 10-fold cross-validation, optimal models for predicting students’ increased interest, post-class study willingness, course satisfaction, and demand for additional TBL courses were identified. The AUC were 0.959 (95% CI: 0.901–1.000), 0.954 (95% CI: 0.895–1.000), 0.948 (95% CI: 0.884–1.000), and 0.927 (95% CI: 0.839–1.000), respectively, with corresponding ROC curves detailed in Fig. 2A, C, E, G. All optimal models achieved AUC values > 0.9, indicating excellent discriminative performance.

**Fig. 3:**
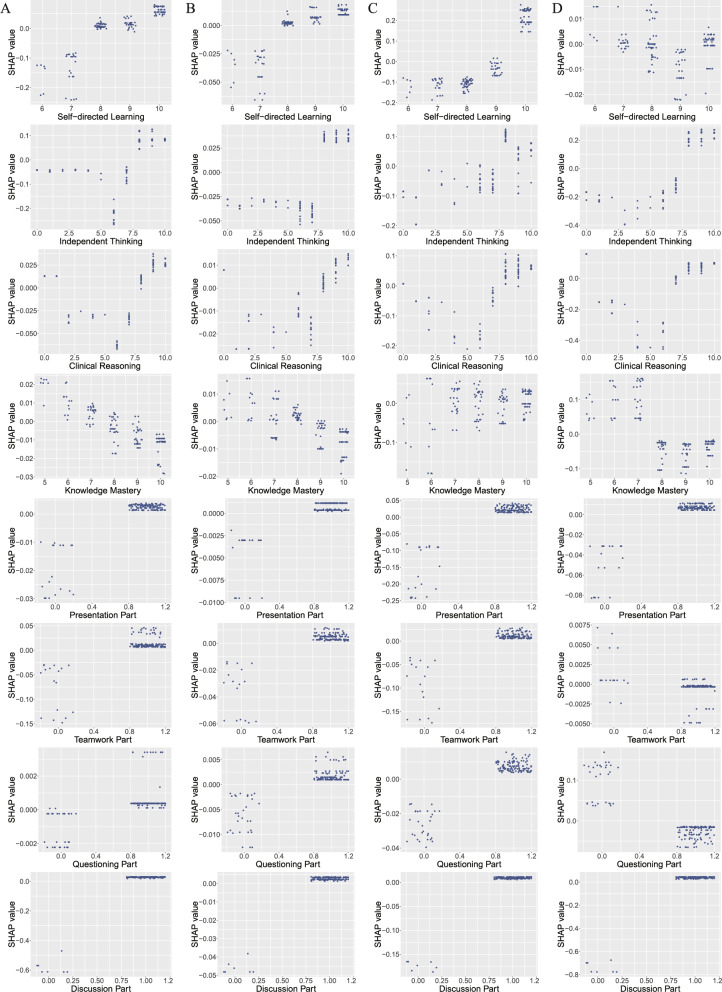
SHAP dependence plots of XGBoost for predicting of students’ overall attitude towards the TBL course (**A**) SHAP dependent plots for each feature of the model predicting whether to enhance student interest; (**B**) SHAP dependent plots for each feature of the model predicting whether students will further learn after class; (**C**) SHAP dependent plots for each feature of the model predicting students’ satisfaction with this course; (**D**) SHAP dependent plots for each feature of the model predicting whether students expect more TBL courses

The SHAP summary plots (Fig. 2B, D, F, H) ordered by descending feature importance revealed that independent thinking capability scores ranked highest in predicting increased interest (mean |SHAP| = 0.0864), after-class studying willingness (mean |SHAP| = 0.0360), and demand for additional TBL courses (mean |SHAP| = 0.2140). This indicates that the independent thinking capability scores is the most significant influence factor when forecasting students’ increased interest, willingness to engage in post-class learning, and demand for additional TBL courses. SHAP dependence plots (Fig. 3) further demonstrated strong positive correlations between these scores and SHAP values: *r* = 0.521 (95% CI = 0.396–0.628) for interest, *r* = 0.716 (95% CI = 0.629–0.785) for study willingness, and *r* = 0.806 (95% CI = 0.742–0.855) for course demand. For course satisfaction prediction, self-directed learning capability scores contributed most significantly (mean |SHAP| = 0.1330), with an exceptionally strong positive correlation (*r* = 0.873, 95% CI = 0.829–0.906). Notably, while recognition of most teaching parts positively correlated with general teaching evaluations, the recognition of the teamwork part (*r* = −0.432, 95% CI = −0.552–−0.284) and questioning part (*r* = −0.900, 95% CI = −0.927–−0.865) showed a negative correlation with the SHAP values for demand for additional TBL courses. Full correlation analysis results are detailed in Table 2.

**Table 2:** Pearson’s correlation of SHAP dependence plots

Model	Feature	*r*	95%CI
Increased Interest	Self-directed Learning	0.849	0.798 to 0.888
Increased Interest	Independent Thinking	0.521	0.396 to 0.628
Increased Interest	Clinical Reasoning	0.484	0.352 to 0.596
Increased Interest	Knowledge Mastery	−0.853	−0.891 to −0.803
Increased Interest	Presentation Part	0.924	0.897 to 0.944
Increased Interest	Teamwork Part	0.830	0.773 to 0.873
Increased Interest	Questioning Part	0.624	0.516 to 0.712
Increased Interest	Discussion Part	0.997	0.995 to 0.997
Afterclass Studying	Self-directed Learning	0.795	0.729 to 0.847
Afterclass Studying	Independent Thinking	0.716	0.629 to 0.785
Afterclass Studying	Clinical Reasoning	0.621	0.513 to 0.709
Afterclass Studying	Knowledge Mastery	−0.677	−0.755 to −0.581
Afterclass Studying	Presentation Part	0.857	0.809 to 0.894
Afterclass Studying	Teamwork Part	0.893	0.856 to 0.921
Afterclass Studying	Questioning Part	0.853	0.803 to 0.891
Afterclass Studying	Discussion Part	0.995	0.994 to 0.997
Course Satisfaction	Self-directed Learning	0.873	0.829 to 0.906
Course Satisfaction	Independent Thinking	0.725	0.640 to 0.792
Course Satisfaction	Clinical Reasoning	0.620	0.501 to 0.701
Course Satisfaction	Knowledge Mastery	0.320	0.170 to 0.455
Course Satisfaction	Presentation Part	0.925	0.899 to 0.945
Course Satisfaction	Teamwork Part	0.883	0.843 to 0.914
Course Satisfaction	Questioning Part	0.954	0.937 to 0.966
Course Satisfaction	Discussion Part	0.998	0.997 to 0.999
More Expectations	Self-directed Learning	−0.226	−0.371 to −0.071
More Expectations	Independent Thinking	0.806	0.742 to 0.855
More Expectations	Clinical Reasoning	0.463	0.329 to 0.579
More Expectations	Knowledge Mastery	−0.686	−0.761 to −0.592
More Expectations	Presentation Part	0.923	0.895 to 0.943
More Expectations	Teamwork Part	−0.432	−0.552 to −0.284
More Expectations	Questioning Part	−0.900	−0.927 to −0.865
More Expectations	Discussion Part	0.998	0.997 to 0.998

The r-value represents the Pearson correlation coefficient between the actual values of a specified feature in a given model and their corresponding SHAP values. A positive r-value indicates a positive correlation, and a negative r-value indicates a negative correlation

## Discussion

The teaching acceptance survey data revealed that over 80% of students significantly increased their interest in cosmetic dermatology through TBL and desired broader adoption of TBL in future courses. Despite the heavy academic workload of undergraduates and TBL’s demanding pre-class preparation requirements, 84.4% of students expressed willingness to continue studying cosmetic dermatology post-class, strongly validating the effectiveness of the interest-oriented instructional design philosophy. However, satisfaction surveys identified areas for improvement: although 86.4% of students desired more TBL courses, process satisfaction reached only 81.2%, indicating that while the novel pedagogical model is widely accepted, adaptation barriers persist among some participants, underscoring the necessity for optimizing teaching components.

Notably, students’ recognition of different TBL parts varied significantly: the discussion part received the highest approval rate (95.5%), while the questioning part garnered only 80.5%. Pedagogical observations suggest this disparity may stem from the following mechanisms: First, the questioning part requires deep engagement, demanding continuous attention to peer presentations and integration of critical thinking, imposing high cognitive load. In contrast, the discussion part’s ambiguous role allocation allows low-intensity participation, facilitating “free rider” phenomena, a common issue in TBL [54, 55]. Second, public questioning may trigger social anxiety, leading students to avoid critical interactions. Thus, TBL refinement should focus on: (1) establishing a structured discussion framework (e.g., explicit role-task lists and quantified contribution metrics) to reduce free-riding via peer evaluation; (2) creating a safe questioning environment (e.g., anonymous inquiry systems) to transform adversarial challenges into constructive academic dialogue.

Enhancing student learning efficacy remains the core objective of pedagogical reform. Existing studies confirm that, compared to traditional LBL, TBL demonstrates superior advantages in cultivating problem analysis skills, self-directed learning capabilities, and lifelong learning competencies [56–58]. This study found that TBL significantly improves students’ self-directed learning and knowledge mastery through preparation for classroom presentations (requiring literature reviews) and intellectual exchange during Q&A sessions (analogous to the “teach” mechanism in the Feynman learning technique) [59]. However, contradicting prior conclusions, students perceived LBL as more effective in fostering independent thinking. Traditionally, LBL is criticized for its teacher-dominated structure, where students passively receive knowledge, a process assumed to hinder critical thinking. This finding suggests that high-achieving learners may still actively construct critical filtering mechanisms during LBL’s unidirectional knowledge transfer rather than passively assimilate information. Additionally, prevalent “free rider” behaviors in TBL may suppress individual critical thinking, explaining why some students prefer the independent learning environment of LBL. The efficacy divergence between pedagogical models arises not only from methodological differences but also from learner characteristics and implementation quality.

In conclusion, the application of TBL in undergraduate cosmetic dermatology education demonstrates significant potential, particularly in stimulating student engagement and enhancing comprehensive competencies. However, it also reveals several challenges. Fortunately, AI technologies offer innovative pathways to overcome these limitations. Machine learning results indicate that enhancing independent thinking capability, which is precisely the core weakness of TBL discovered by our study, is a critical factor for improving students’ disciplinary interest in cosmetic dermatology and their acceptance of TBL. Therefore, future TBL pedagogy must emphasize balancing the cultivation of teamwork collaboration and independent decision-making skills, particularly in clinical practice, where physicians are often required to independently execute critical clinical decisions.

Machine learning also uncovered a critical paradox: while improvements in knowledge mastery, which is students’ primary motivation for participation, positively correlated with course satisfaction, they exhibited significant negative correlations with deepened interest in cosmetic dermatology, post-class study willingness, and acceptance of the TBL. This finding highlights a pivotal challenge in educational research: traditional TBL, despite effectively enhancing academic performance through intricate process designs (e.g., multi-stage testing and case discussions), may erode intrinsic learning motivation due to hidden learning burdens (e.g., excessive pre-class preparation time and ambiguous role allocation) [60, 61]. Notably, this paradox became explicit only through machine learning-based feature importance analysis, underscoring the need for educational evaluation to deeply analyze cognitive load distribution during instruction, rather than solely focus on improvements in academic performance. This necessitates educators adopting more flexible adaptive strategies when implementing TBL, by streamlining redundant steps, establishing transparent contribution assessment mechanisms, and integrating engaging thematic modules, while preserving its core strengths (team collaboration, clinical thinking cultivation), to achieve dynamic equilibrium between knowledge acquisition and motivational sustainability. Pedagogical refinement should avoid mechanically replicating successful workflows from other disciplines; instead, it requires localized redesign based on disciplinary characteristics and student cognitive profiles, ensuring TBL evolves into an effective tool for nurturing multidimensional competencies [62].

While students’ recognition of individual teaching parts generally positively predicted general teaching evaluations, a paradoxical phenomenon emerged in assessing demand for additional TBL courses: students who endorsed the teamwork part and questioning part exhibited reservations toward expanding TBL implementation, whereas those who did not endorse these components paradoxically showed higher acceptance. Nevertheless, this paradoxical finding aligns with our results obtained through traditional statistical methods, which indicated that the questioning part was the most resisted component of TBL among students. This inverse relationship reveals latent pedagogical tensions: although students did not explicitly oppose specific component designs, subconscious cumulative burnout, stemming from task overload in teamwork (e.g., redundant work due to ambiguous role allocation) and the adversarial potential of questioning sessions (e.g., social anxiety from public inquiries), translated into systemic resistance to TBL. Educators must confront this cognitive dissonance between “instructional design” and “learning experience”: while introducing TBL to peripheral disciplines like cosmetic dermatology aims to ignite disciplinary interest and cultivate dedicated professionals, practice inadvertently reinforces knowledge-centric orientations, leading to latent stress accumulation. This misalignment between intent and practice echoes the “intention-action gap” in education [63, 64]. In contrast to traditional methods that rely on human interpretation of statistical results, AI demonstrates greater utility in identifying these underlying “intention-action gap”.

A key innovation of this study lies in introducing interpretable supervised machine learning to analyze TBL teaching efficacy. Compared to unsupervised generative AI used in prior research, this approach significantly enhances result credibility and stability [65, 66]. SHAP-based interpretability analysis not only accurately identifies critical pedagogical determinants (e.g., enhancing independent thinking) but also reveals anomalies easily overlooked by traditional methods (e.g., the negative association between high knowledge mastery and low learning interest, and the inverse link between high teach part recognition and low demand for course expansion). This methodological advancement provides robust evidence-based guidance for TBL refinement. The anomalous phenomena identified in this study challenge the prevailing assumption that interactive pedagogical methods like TBL and PBL are inherently superior to traditional LBL approaches. In reality, these emerging instructional models still contain multiple aspects requiring refinement. The methodological advancements employed in this research not only provide a novel perspective for objectively evaluating teaching effectiveness but also deliver crucial evidence-based foundations for the systematic assessment and precise optimization of TBL methodology.

This study has several limitations. First, as an exploratory trial, the single-center design and limited sample size of this study may affect the generalizability and external validity of the findings. Future studies should incorporate additional research centers and implement TBL teaching practices across diverse topics and educational settings. By systematically collecting multi-source teaching data to expand the modeling sample size, the robustness and generalizability of the research conclusions can be further enhanced. Second, this study predominantly centered on the comparison between TBL and LBL, without incorporating other interactive pedagogical approaches such as PBL or CBL. Future research should systematically compare TBL with a broader range of innovative teaching methods, aiming to elucidate the distinctive characteristics and applicable contexts of different interactive instructional models. Such comparative work will provide a more comprehensive empirical foundation for the optimization of teaching strategies. Finally, it should be noted that the IML approach adopted in this study presents a higher technical threshold for application compared to traditional statistical methods and generative AI technologies. Its analytical process necessitates involvement of specialists in machine learning, which currently constitutes one of the key limiting factors for its broader adoption in the field of education.

## Conclusions

This study successfully implemented TBL in undergraduate cosmetic dermatology education and systematically compared it with traditional LBL. By innovatively introducing IML, it provided an in-depth analysis of TBL’s core advantages and potential limitations, clearly identified the approach’s constraints at the current stage, and proposed targeted refinement strategies. The research further revealed a misalignment between educational theory and practical outcomes: although TBL’s original intent is to stimulate disciplinary interest and cultivate professional competence, its implementation may inadvertently reinforce a knowledge-centered teaching orientation. This discrepancy between objectives and practice demonstrates that no single teaching method is universally optimal. Establishing effective teaching monitoring and dynamic adjustment mechanisms is crucial for ensuring that instructional approaches consistently serve the fundamental goal of talent development.

TBL demonstrates significant value in peripheral disciplines like cosmetic dermatology, where class hours are limited and examination weight is low. By synergizing in-class and extracurricular activities, TBL fully mobilizes student agency, enhancing teaching efficiency per unit time and amplifying the impact of constrained schedules, thereby boosting the discipline’s professional appeal. However, based on the findings of this study, several key challenges confronting TBL pedagogy have been identified, including but not limited to excessive reliance on teamwork, lack of individual decision-making opportunities, and the “free rider” phenomenon. These issues require attention and targeted measures for improvement. Future improvements should focus on: (1) establishing explicit role allocation rules and incentive mechanisms to strengthen independent thinking through task accountability and outcome traceability; (2) optimizing interactive formats to reduce adversarial pressure. For instance, pre-discussions within groups could culminate in unified viewpoints presented by designated representatives, or anonymous text submissions could enable instructor-mediated academic debates, preserving both student privacy and intellectual rigor.

This study, utilizing AI methodologies, reveals the complex interrelationships among stimulating learning interest, developing comprehensive competencies, and improving academic performance. This finding presents new requirements for educators: teaching strategies must be dynamically adapted based on specific educational objectives and contextual characteristics to achieve continuous optimization of the teaching process. Furthermore, the scientific validity and effectiveness of teaching optimization should not rely solely on experiential judgment but must fully leverage objective data analytics driven by AI (e.g., feature importance ranking, anomaly correlation identification) to construct a data-driven, evidence-based teaching decision-making system. By developing intelligent models linking pedagogical elements to learning outcomes, we can deeply dissect various teaching methods (e.g., TBL, PBL) to inform evidence-based personalized refinements. This AI-driven educational analytics approach not only offers novel perspectives for dermatology training but also holds significant potential for broader application in teaching evaluation and systematic refinement across all clinical disciplines, thereby advancing medical education toward more precise, interpretable, and systematic development.

## Data Availability

The data that supported this study are available upon request from the corresponding author.
